# The Illegitimate Child

**Published:** 1917-07-14

**Authors:** 


### The Illegitimate Child.

The question of the illegitimate child and its mother was discussed on July 5 under the chairmanship of the Bishop of Kensington. Judge Neil put forcibly the point that the life of the respectable wife and mother of the poorer classes needs to be made better worth living. Much was said on the question of the feeble-minded and their children. The benevolent helper can often see that responsibility for a child will draw out the best in a very weak nature. On the other hand, Mr. Gascoigne Hartley laid stress on the immediate harm a mother may do to her child while her own character is being established. The prohibition to midwives working under nursing associations to attend such cases was warmly discussed, with its corollary in the frequent practice of sending for the nurse when the girl is actually in labour, when she *must* remain. Miss Burnside touched on the painful fact that the nurse is not forbidden to attend a married woman who is seriously ill through procuring abortion. It was urged that better laws regarding adoption are needed, and especially to avoid the possibilities of blackmail, also that the refusal of many maternity homes to take in disgraced girls had sometimes serious effects on younger sisters, from whose company in the house the mother-to-be would be better removed. The afternoon audience had reason to be grateful to Mrs. Bramwell Booth for her wise and weighty words, and the high moral plane of all her arguments. The terrible mortality of the illegitimate, twice, or, according to Dr. Sloan Chesser, three times that of the legitimate, was such as the country would not permit if lambs were in question. Responsibility must be made to touch the father in every possible case, *not* by the mother, but by the State. Marriage should legitimise a child as in Russia and elsewhere, the fact that it does not often causes a girl to refuse to marry the father of her child, and prefer to bear her life burden alone. In some cases a less costly divorce would make marriage possible; the cheapest she had known cost £8 8s. to people who afforded with great self-denial this effort to do right. In the discussion that followed, the significant fact was elicited that a new baby farming is in progress. An advertisement in a paper of the highest position brought thirty-nine replies; thirty-seven offered to take over the child for a premium varying from £20 to £500. Mr. Mansel Moullin proposed a resolution, which was unanimously carried, to the effect that (1) marriage should legitimise the child, (2) the onus of securing its maintenance from the father should be laid on the local authority, (3) that the authority should have the power to increase the allowance as may appear fit. In the papers and discussion on children under the Poor Law, the same points emerged again, the desire to establish every child as a self-respecting citizen is hopefully growing, and promises well for a reduction of our vast expenses in preventive and punitive institutions.

